# Epidemiology, Translation and Clinical Research of Ophthalmology

**DOI:** 10.3390/jcm12113819

**Published:** 2023-06-02

**Authors:** Kai Jin, Wenyue Shen, Yuanbo Liang, Mingguang He

**Affiliations:** 1 Eye Center, The Second Affiliated Hospital School of Medicine Zhejiang University, Zhejiang Provincial Key Laboratory of Ophthalmology, Zhejiang Provincial Clinical Research Center for Eye Diseases, Zhejiang Provincial Engineering Institute on Eye Diseases, Hangzhou 310009, China; shenwenyue2000@163.com; 2 National Clinical Research Center for Ocular Diseases, Eye Hospital of Wenzhou Medical University, Wenzhou 325027, China; yuanboliang@126.com; 3 Centre for Eye Research Australia, Royal Victorian, Eye and Ear Hospital, University of Melbourne, Melbourne 3002, Australia

The human eye is a complex and vital organ that plays a significant role in maintaining a high quality of human life. Impairment or damage to human eyes can cause negative health effects, resulting in increased morbidity, mortality, and socioeconomic burdens [1,2]. Cataract, undercorrected refractive error, and glaucoma are among the leading causes of blindness and moderate to severe vision impairment (MSVI) globally [3]. The demand for eye care services is expected to increase due to the aging and growing world population, and early detection and timely intervention will be essential to reduce morbidity from these conditions in the future [3].

Recent advances in non-invasive ophthalmic imaging technologies, such as optical coherence tomography (OCT), OCT angiography (OCTA), specular and confocal microscopy, widefield imaging, and ultrasonography, etc. [4,5,6,7,8], have revolutionized the diagnosis, evaluation, management, and monitoring of various eye diseases. Other emerging imaging technologies, including fluorescence lifetime imaging ophthalmoscopy (FLIO) [9], retinal oximetry and hyperspectral imaging (HSI) [10], artificial intelligence (AI) approaches [11], and smartphone-based fundus imaging (SBFI), have also gained increasing interest in recent years [12]. With the development of these medical imaging technologies as well as the improvement of disease treatment methods, the scientific community’s understanding of ophthalmic diseases is gradually deepening.

Among f the advantages of ophthalmic imaging technologies is the ability to observe nerves and blood vessels within the eye in great detail. The transparency of the eye’s refractive medium allows for clear visualization, and these imaging techniques provide us with high spatial resolution and contrast images at a relatively low cost and with wide availability. These images offer detailed information about the function and morphology of the eye and play an indispensable role in diagnosis, assessment, management and monitoring of various ocular conditions [4,8,13].

The early prevention, timely diagnosis, and effective treatment of eye diseases can alleviate the public health burden caused by visual impairment. Various imaging modalities can serve as effective screening tools or predictors to detect possible eye diseases that require comprehensive ophthalmic examinations and treatments to circumvent visual impairment and even blindness. For instance, diabetic retinopathy [14,15], glaucoma [16], age-related macular degeneration (ARMD) [17], and myopia [18] are some of the conditions that can be detected via the use of imaging techniques.

In addition to detecting eye diseases, advances in ophthalmology provide clues for systemic diseases or neurological disorders. Retinal fundus images have been utilized to train deep learning models to predict several cardiovascular risks, a technology with applications to screening for diabetic eye disease [19]. OCTA can detect changes in retinal or choroidal microcirculations, allowing for the diagnosis, monitoring, and prognosis stratification of systemic diseases such as systemic lupus erythematosus (SLE) [20] and systemic sclerosis (SSc) [21]. Several retinal imaging techniques (e.g., OCT, OCTA and digital retinal photography) can be used to study the neuronal and vascular structures of the retina in patients with Alzheimer’s disease (AD); these can be combined with computerised algorithms in the early detection and screening of AD [22].

This Special Issue is dedicated to ophthalmology research, with a focus on epidemiology, translational research, and clinical studies that cover the entire visual system, including the cornea, lens, vitreum, retina, choroid, and optic nerve. Various research methods are welcome. These include, but are not limited to, statistical methods, artificial intelligence, and biology experiments.

We welcome descriptive or analytical studies from the field of epidemiology that address the prevalence, risk factors, and preventive measures of eye diseases. Moreover, we encourage authors to contribute studies that utilise ophthalmic information as novel biomarkers of systemic disease. Translational research that aims to apply novel screening, diagnostic, and therapeutic technologies to clinical practice is also highly encouraged.

In terms of clinical research, we welcome randomized or non-randomized controlled studies that focus on ophthalmic diseases. In addition, secondary research based on evidence analysis of published clinical evidence, such as meta-analysis, is also welcome.

We invite informative reviews and innovative research articles that will lead to a deeper understanding of this issue, better translation, and more evidence-based evidence for ophthalmic diseases. We look forward to receiving your submission to this Special Issue.
